# Problem areas in diabetes and glicemic control in type 1 diabetes in a public diabetes center

**DOI:** 10.1186/1758-5996-7-S1-A168

**Published:** 2015-11-11

**Authors:** Paula Lamego Lourenço, Sônia Maria do Carmo Maulais, Maria Eugênia Sllva Hitchon, Janice Sepúlveda Reis

**Affiliations:** 1 Centro de Especialidades Médicas (CEM) da Santa Casa de Belo Horizonte, Belo Horizonte, Brazil

## Background

People with type 1 diabetes (T1D) encounter a series of chronic stressors related to their condition. Common issues include worry about complications, fear of hypoglycemia, and inescapable preoccupation with food, exercise, and dietary regimens. Other sources of distress include misplaced illness beliefs, lack of knowledge, social support or explanations as well as feelings of being overwhelmed by the illness and its requirements.

## Objective

Assess emotional problems related to diabetes in relation to glycemic control in patients followed in an Educational Program of a public service.

## Materials and methods

Seventy-seven patients diagnosed with T1D followed for a minimum period of one year in interdisciplinary treatment and education program were divided into two groups according to A1C (<8%, n=40 and ≥ 8%, n=37). Structured interview and the instrument B-PAID (Brazilian version of the PAID scale-Problems Areas in Diabetes) were performed individually with each participant.

## Results

In both groups were found B-PAID total score <40 points, which means low level of emotional stress. In the comparison between the groups was demonstrated score significantly greater in group with A1C ≥ 8% (p <0.05) within the sub-dimensions related to emotional (p 0.01) treatment (p 0.042) and social support (p 0.009). Age correlated negatively with social support (p 0.039). The A1C was positively correlated with the total score on the B-PAID (p 0.026) and the sub-dimensions related to emotional (p 0.044), treatment (p 0.016) and social support (0.001).

## Conclusions

In people with T1D, DM-specific distress measured by the PAID correlated significantly with impaired glycemic control, even in patients regularly treated in a diabetes center with formal diabetes education. Specific educational programs aimed to these groups could help in achieving glycemic targets.

